# Pitfalls of Thrombotic Microangiopathies in Children: Two Case Reports and Literature Review

**DOI:** 10.3390/diagnostics13071228

**Published:** 2023-03-24

**Authors:** Adriana Mocanu, Roxana Alexandra Bogos, Tudor Ilie Lazaruc, Anca Lavinia Cianga, Vasile Valeriu Lupu, Ileana Ioniuc, Mirabela Alecsa, Ancuta Lupu, Anca Viorica Ivanov, Ingrith Crenguta Miron, Iuliana Magdalena Starcea

**Affiliations:** 1 Mother and Child Medicine Department, Discipline of Pediatrics, “Grigore T. Popa” University of Medicine and Pharmacy, 16 Universitatii Street, 700115 Iasi, Romania; 2 Nephrology Division, St. Mary’s Emergency Children Hospital, 700309 Iasi, Romania

**Keywords:** thrombotic microangiopathy, atypical hemolytic-uremic syndrome, thrombotic thrombocytopenic purpura, COVID-19

## Abstract

Thrombotic microangiopathy can present itself in the form of several clinical entities, representing a real challenge for diagnosis and treatment in pediatric practice. Our article aims to explore the evolution of two rare cases of pediatric thrombotic thrombocytopenic purpura (TTP) and atypical hemolytic uremic syndrome (aHUS) with extremely similar clinical pictures, which, coincidentally, presented at approximately the same time in our hospital. These cases and our literature review demonstrate the multiple facets of thrombotic microangiopathy, which can produce various determinations and salient manifestations even among the pediatric population. TTP and aHUS may represent genuine diagnostic pitfalls through the overlap of their clinical and biological findings, although they develop through fundamentally different mechanisms that require different therapeutic approaches. As a novelty, we underline that COVID-19 infection cannot be excluded as potential trigger for TTP and aHUS in our patients and we predict that other reports of such an association will follow, raising a complex question of COVID-19’s implication in the occurrence and evolution of thrombotic microangiopathies. On this matter, we conducted literature research that resulted in 15 cases of COVID-19 pediatric infections associated with either TTP or aHUS. Taking into consideration the morbidity associated with TTP and aHUS, an elaborate differential diagnosis and prompt intervention are of the essence.

## 1. Introduction

Microangiopathic hemolytic anemia (MAHA) represents a group of disorders characterized by hemolytic anemia along with thrombocytopenia and thrombi formation in different organs, comprising two main pathological entities: thrombotic thrombocytopenic purpura (TTP) and hemolytic uremic syndrome (HUS). TTP is a disorder characterized by the formation of platelet-rich thrombi within the vasculature, resulting from a severe deficiency of the von Willebrand factor (vWF)-cleaving metalloproteinase, ADAMTS13 [1]. TTP represents a rare and severe medical condition, especially in the pediatric population. The average annual prevalence of this condition is 10 cases per 1 million individuals, and it is associated with a high mortality rate, estimated to be between 10% and 20% [2]. The timely recognition and diagnosis of TTP is essential for the effective management of this condition and improving the patient’s prognosis. In clinical practice, the first-line treatment for TTP rests on plasma exchange therapy (PEX) [3,4]. Atypical hemolytic and uremic syndrome (aHUS) is a type of thrombotic microangiopathy that is caused by dysregulation of complement activation, either genetic or acquired [5]. A recent report suggests that novel coronavirus 2 (SARS-CoV-2) infection may act as a trigger for atypical hemolytic uremic syndrome [5,6,7]. Severe COVID-19 leads to widespread microvascular damage, which includes thrombotic microangiopathy. This damage is primarily caused by inflammation, cytokine storm, and dysregulated complement activation. Soluble C5a and C5b-9, which are biomarkers of alternative and terminal complement pathway activation, are found circulating in the bloodstream, and their levels correlate with the severity of COVID-19 infection in children [8]. SARS-CoV-2 infection has been linked with the development of several autoimmune diseases such as thrombotic thrombocytopenic purpura, immune thrombocytopenic purpura, autoimmune hemolytic anemia, or systemic lupus erythematosus [9].

## 2. Materials and Methods

### 2.1. Case Reports

The present article reviews the clinical presentation, pathophysiology, differential diagnosis, treatment and evolution of thrombotic microangiopathies in a case of TTP and one case of aHUS, both in pediatric patients with a history of SARS-CoV-2 infection. 

This is a retrospective study, approved by the IRB, on two cases of thrombotic microangiopathy, with patients hospitalized in Pediatric Nephrology and Pediatric Onco-Hematology Clinics of “St. Mary” Children’s Emergency Hospital, Jassy, Romania.

The research adhered to the principles of the Declaration of Helsinki and was approved by the Ethics Committee (6878/26.02.2022) of our hospital.

### 2.2. Literature Review and Data Extraction

A search was conducted on the PubMed database to retrieve all the published literature on pediatric atypical hemolytic uremic syndrome and thrombotic thrombocytopenic purpura. The Medical Subject Headings (MESH) terms we used were (((atypical hemolytic uremic syndrome [Mesh Terms]) OR (aHUS [Mesh Terms])) AND (thrombotic thrombocytopenic purpura [Mesh Terms])) OR (immune thrombocytopenic purpura [Mesh Terms]) AND (pediatric [Mesh Terms]) OR (paediatric [Mesh Terms])) AND (((COVID-19 [Mesh Terms]) OR (SARS-CoV-2 infection [Mesh Terms])). The search did not have any limitations on the type of article or publication date. After removing duplicate articles and those without access to full-text reports, 9 case reports were identified, regardless of language, which included pediatric patients diagnosed with aHUS and TTP in relation to COVID-19 infection. The following data retrieved from each report are presented in Table S1: (1) first author’s name and year of publication, (2) underlying condition, (3) sex and age of the patient, (4) significant laboratory characteristics, (5) ADAMTS13 activity and antibodies, if reported, (6) aHUS underlying mechanism, (7) COVID-19 antibody titer, (8) main clinical features, (7) treatment, and (8) patient evolution. The search was conducted in February 2023.

## 3. Results

### 3.1. Case 1—aHUS

A two-year-old boy has been referred to our clinic for severe diarrhea (10 stools per day) starting 4 days before presentation, with tonic–clonic seizures, oligo-anuria and melenic stools installing in evolution. Clinical examination showed profound general distress, severe dehydration, tachycardia, blood pressure 104/55 mmHg, systolic heart murmur, normal lung auscultation and no justification indicated for an acute surgical intervention for abdomen, while maintaining melenic diarrheal stools and anuria. CBC count revealed normochromic normocytic anemia (hemoglobin 9.3 g/dL) and thrombocytopenia (39,000/mmc) while renal function assessment confirmed a severe acute kidney failure (AKI) (creatinine 3.9 mg/dL, blood urea nitrogen = 185 mg/dL) corresponding to class III pRIFLE AKI. A clinical suspicion of a thrombotic microangiopathic anemia was confirmed trough peripheral blood smear showing 8% schistocytes and increased LDH in context of hemolysis. Stool cultures were negative for Shiga-toxin producing *E. coli* serotypes. Other biological findings are shown in Table 1.

**Figure 1:**
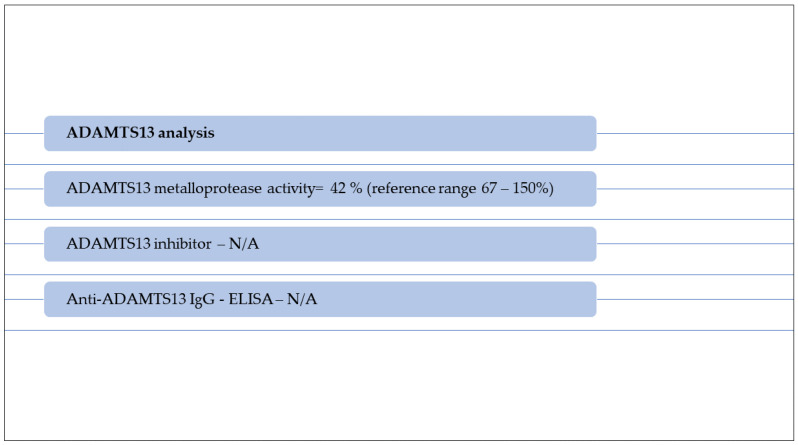
ADAMTS13 analysis in a patient with suspected aHUS.

**Figure 2:**
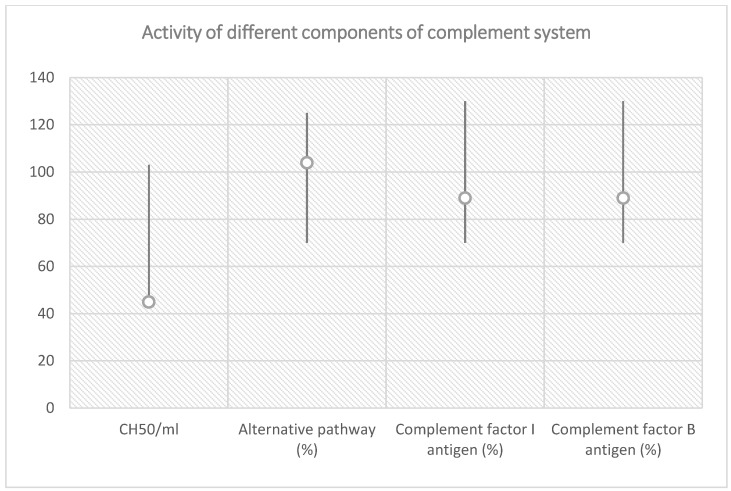
Classical pathway, alternative pathway, complement factor I and factor B antigen activity in relation with reference range.

Rehydration and large spectrum antibiotic therapy (carbapenem, linezolid, metronidazole) were initiated with immediate effect. A decrease in hemoglobin, thrombocytes number and renal function followed, with consistent anuria, hypervolemia, hypertension and unilateral lower limb myoclonic seizures, accompanied by signs of mild cerebral edema, bilateral ethmoiditis, left lung infiltration and bilateral pleurisy on computed tomography. Hemodiafiltration, antihypertensive treatment (calcium-blocking agents, central alfa-dilators) and blood transfusions were started. Facing a TMA in a two-year-old boy with no prior disease history, aHUS was suspected and plasma exchange therapy was initiated. Serology for hepatic viruses B and C, immunodeficiency virus, Epstein–Barr virus, cytomegalovirus, Toxoplasma and COVID-19 PCR were negative. Serology for COVID-19 was also performed, showing moderately increased titers of anti-Spike protein IgG subunit S1 (340 UI/L) and S2 (399 UI/L); therefore, a decision of implementing i.v. immunoglobulin administration was made. Blood sampling for biochemical and molecular analysis of complement system was carried out before treatment in the Research Laboratory of Semmelweis’ University, Department of Internal Medicine and Hematology, Hungary. Results are shown in Figure 1 and Table 2, while a graphic representation of classical pathway alternative pathway, factor I and factor B antigen activity are shown in Figure 2. On their basis, TTP was excluded (ADAMTS13 activity decreased, but was not deficient), and ongoing hemolysis was noted (severely decreased haptoglobin). The results are indicative of classical pathway activation and consumption, therefore potentially related to triggering infection. In context of the absence of signs of amelioration despite advanced supportive measures including plasmapheresis and i.v. immunoglobulin, inhibitory complement therapy (Eculizumab) was started. A dose of 600 mg was administered, repeated at one week, with concomitant anti-meningococcal and *H. influenzae* vaccination. Recovering of diuresis, renal function amelioration, a decrease in blood pressure and increase in hemoglobin and thrombocytes number slowly installed, confirming a complement-mediated TMA in our patient, specifically aHUS. In this context, the patient was initiated on a therapeutic protocol for aHUS consisting in administration of Eculizumab 300 mg every 2 weeks. During the latest follow-up visit, clinical examination was normal. He presented with Hb 10 g/dL, with normal LDH, less than 4% schistocytes on peripheral blood smear, no thrombocytopenia, creatinine 0.58 mg/dL with a renal clearance (Schwartz pediatric formula) of 73 mL/min/1.73, normal C3 and C4 fractions with no proteinuria. Administration of anti-C5 monoclonal antibody had to be continued as an underlying genetic mutation in the complement system has not yet been ruled out.

### 3.2. Case 2—TTP

A 16-year-old Romanian female presented to the Saint Mary Emergency Children Hospital from Jassy on 5 January 2023, complaining of asthenia, diffuse headache, odynophagia, dysphagia, fever, vomiting and a semi-solid stool, symptoms that started approximately 10 days before presentation.

The history of the patient revealed that she is known to have iron-deficiency anemia for which she recently (November 2022) underwent iron replenishment treatment at home. No recent vaccinations were observed. The patient did not have any significant medical history, nor did she report any tobacco, alcohol or drug abuse. She had not received a blood transfusion in the past and was not taking any medications at the time of admission. Additionally, there was no notable family medical history. Physical examination revealed an afebrile, normotensive female with headaches, marked asthenia, pale, subicteric skin and mucosa, no petechia, no bleeding, discrete pharyngeal congestion observed. The patient did not exhibit any signs of splenomegaly, liver hypertrophy, or lymph node hypertrophy upon physical examination. There were no other noteworthy observations made during the examination. Abdominal ultrasound and echocardiogram did not reveal any significant findings, and the urine pregnancy test came back negative. The complete blood cell count (CBC) revealed WBC = 11,510/mmc, severe thrombocytopenia = 9000/mmc, severe hemolytic anemia with Hb = 4.7 g/dL, MCV = 92.3 fL, reticulocytosis = 14.23%, increased LDH = 1837 U/L, hyperbilirubinemia due to the indirect fraction, increased ferritin = 2269.82 µg/L, and haptoglobin was <25 mg/dL. D-dimer level was 3578 ng/mL, there was no inflammatory syndrome, normal liver and kidney functions (creatinine = 0.6 mg/dL), APTT = 24.7 s, INR = 1.16, Fibrinogen 355.8 mg/dL, PT = 84.2%. Normal immunogram, normal triglycerides. 

ASLO < 50 U/mL. Complement C3 and C4 within normal limits. Serum iron, amylase, total proteins, ionogram came out within normal limits. Urinalysis showed hemoglobinuria and proteinuria. Blood smear showed erythroblasts = 6/100 elements, hypochromia, anisopoikilocytosis (microcytes, normocytes, schistocytes, spherocytes, macrocytes, dacryocytes), platelets in low numbers. Schistocytes 3–4%.

Bone marrow biopsy revealed myelocyte = 1%, metamyelocyte = 4%, polymorphonuclears = 67%, monocytes = 4%, lymphocytes = 24%, erythroblasts 8%, anisopoikilocytosis (megalocytes, schistocytes), platelets in very low number. Immunophenotyping identified 0.6% myeloid precursors, with phenotype CD45+int* CD34+/− CD117+ cyMPO−/+ HLADR+, CD13+ CD38+. All other markers whose expression was evaluated were absent. In addition, 0.5% B lymphoid precursors (with CD34− phenotype- cyCD79a+ CD19+) were described. In the same sample, 48% of cells of the granulocytic series (of which 0.65% were eosinophils) were also identified: 2.2% monocytic series cells, 43.5% cells of the erythroid series, 4.4% lymphocytes, 0.4% plasma cells. The examination was completed by evaluating the thyroid function, which was normal. The anti-nuclear antibody (ANA) test was within normal range, while the extractable nuclear antibody profile showed a positive antibody (ANA)-HEp-2 test. The direct Coombs’ test came out positive, while antineutrophil cytoplasmic antibodies, anti-double-stranded deoxyribonucleic acid (dsDNA) antibodies, lupus anticoagulants, anti-β2-glycoprotein antibodies, anticardiolipin antibodies, and anti-red blood cell antibodies were negative.

The tests conducted to detect the presence of various pathogens such as human immunodeficiency virus (HIV), hepatitis C virus, hepatitis B virus, Epstein–Barr virus, cytomegalovirus, influenza virus or Mycoplasma *pneumoniae* were negative. While RT-PCR RNA-SARS-CoV-2 was also negative, elevated titers of SARS-CoV-2 IgM and IgG were detected. In the face of hemolytic anemia and severe thrombocytopenia, blood transfusion was initially indicated. Six hours after the admission, PLASMIC score was calculated with a result of six points, indicating a high risk of TTP (Table 3).

The PLASMIC score is a clinical prediction tool consisting of seven components that have been designed to accurately determine the pretest probability of severe ADAMTS13 deficiency (*C* statistic 0.96, with a 95% confidence interval 0.92–0.98) [10,11]. This evaluation was completed by calculating the French TMA, which revealed a score of two, indicating a high risk of TTP for our patient (Table 4).

**Figure 3:**
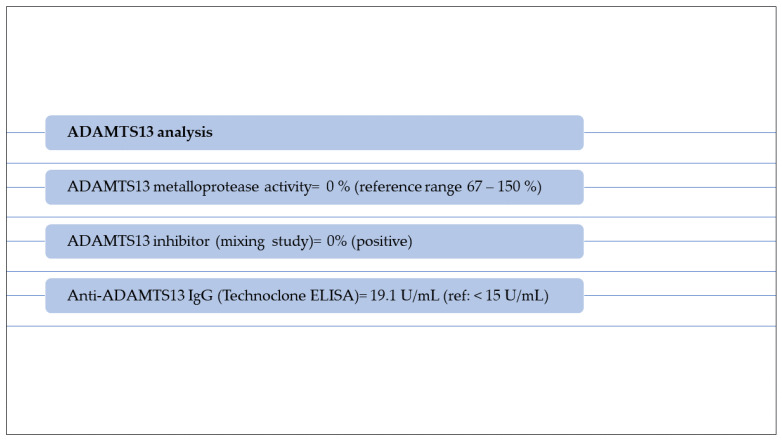
ADAMTS13 analysis in a patient suspected with TTP.

**Figure 4:**
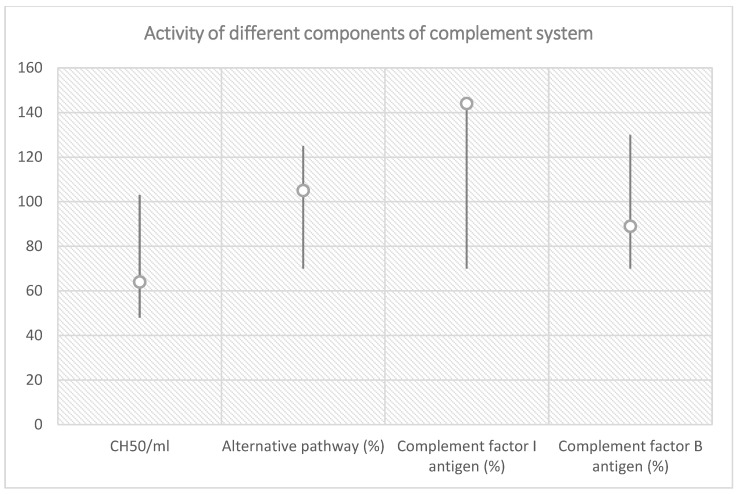
Classical pathway, alternative pathway, complement factor I and factor B antigen activity in relation with reference range.

Consequently, TTP was suspected. Therefore, a biological sample was collected and the ADAMTS13 activity was analyzed qualitatively, which revealed absent activity (0%). Before initiating the treatment, multiple plasma samples were collected and analyzed in the Research Laboratory of Semmelweis’ University, Department of Internal Medicine and Hematology, Hungary. The investigations results are shown in Figure 3 and Table 5, while a graphic representation of classical pathway alternative pathway, factor I and factor B antigen’s activity are shown in Figure 4.

These results reconfirmed that the ADAMTS13 was deficient, supporting the diagnosis of TTP. Haptoglobin level was severely decreased, indicating the presence of intravascular hemolysis.

Subsequently, the patient’s results for the ADAMTS13 inhibitor returned positive, hence the diagnosis of immune TTP was verified. Treatment for the patient involved six cycles of PEX and corticosteroids resulting in a normalized platelet count and reduction in schistocytes to 1–2%. Throughout her hospital stay, the patient did not display any signs of thrombosis, and all vascular ultrasounds showed no signs of modifications. Upon discharge, the patient was prescribed tapered oral methylprednisolone; at the last follow-up visit, the patient’s CBC maintained normalcy, and the clinical examination showed no abnormalities.

### 3.3. Literature Research

Our literature research revealed nine case report-styled articles [12,13,14,15,16,17,18,19,20] comprising 15 pediatric cases of MAHA related to COVID-19 infections, of which six patients were diagnosed with TTP and nine patients were diagnosed with aHUS. Four out of six patients in TTP group were females, while six out of nine subjects in aHUS group were males. Median age at presentation for TTP group was 15.5, in contrast to 7 for aHUS patients. One patient in TTP group eventually expired, while one patient in aHUS group has not recovered renal function, requiring chronic hemodialysis. All other cases had an evolution towards full or partial resolution. All these patients presented with schistocytes on peripheral blood smear and high LDH serum levels, as proof for MAHA. Clinical features, treatment modalities and other characteristics of these patients are shown in Table S1 (in Supplementary Files).

## 4. Discussion

### 4.1. aHUS

**Figure 5:**
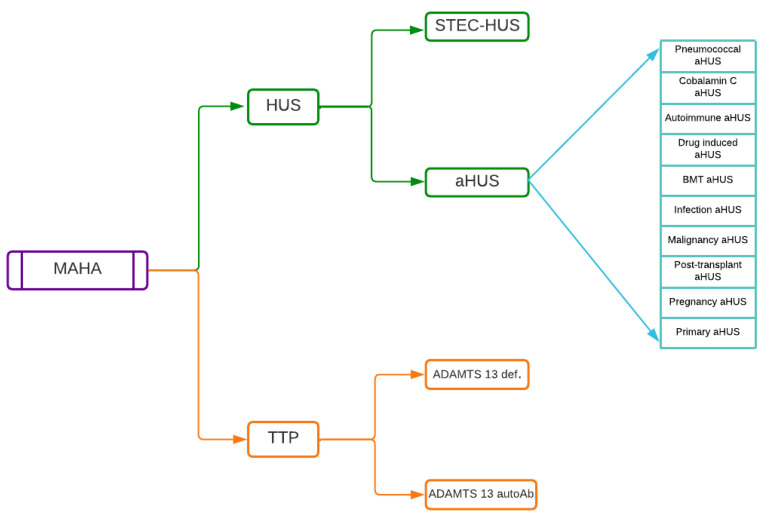
Pathological entities in differential diagnosis of MAHA. After Goodship et al., 2017 [21], MAHA—Microangiopathic hemolytic anemia; HUS—hemolytic uremic syndrome; STEC—Shiga-Toxin *E. coli*; aHUS—atypical hemolytic uremic syndrome; BMT—bone marrow transplant; TTP—thrombotic thromboangiopathic purpura.

In 2017, Goodship et al. [21] proposed a classification of TMA comprising TTP, HUS related to Shiga-toxin producing *E. coli* (STEC-HUS) and atypical HUS, multiple subgroups being described in the latter, as shown in Figure 5. This classification underlines the fact that a diagnosis of primary aHUS can only be made after excluding numerous etiologies such as bacterial and viral infections, autoimmune disease, drug-induced aHUS, malignancy and others. In this regard, a confusing factor is that in the literature, the term “aHUS“ is frequently used only to describe hemolytic uremic syndrome without coexisting disease, i.e., primary aHUS. Nevertheless, there is at least some consensus that historic terminology describing diarrhea-positive (D+) and diarrhea-negative (D+) HUS should not be used anymore, since there are multiple patients with complement-mediated aHUS who present with diarrhea or colitis [22,23], a fact also confirmed by our clinical case. Here, we refer to aHUS according to Goodhips’ et al. classification.

### 4.2. Clinical and Molecular Diagnosis

MAHA, a low platelet count and renal failure, are the clinical criteria supportive of aHUS, with neurological findings and fever being less common in such patients. This is in contrast to our clinical case, in which tonic–clonic seizures were present early in the beginning. However, from a pathophysiological point of view, thrombi formation in any organ can occur. Clinical criteria might not all be present at the same time; therefore, physical and biological evaluation at onset and in evolution provides the most important arguments for a positive diagnosis. Negative testing both for ADAMTS13 deficiency or presence of anti-ADAMTS13 autoantibodies, with a negative assessment for STEC-HUS trough Shiga-like toxin testing and STEC stool culture, supports the diagnosis of aHUS, for which further secondary causes must be ruled out in order to verify the diagnosis of primary aHUS.

Complement system is the primary focus in pathophysiology of aHUS, as opposed to TTP. In aHUS, alternative pathway dysregulation seems to be implicated in more than 60% of cases [23,24,25] with possible findings in complement pathways exploration being decreased in Factor H or Factor I—main regulators of complement system—decreased C3 with normal or moderately decreased C4, anti-Factor H antibodies or no identifiable biochemical modifications in complement system exploration [22].

Important genes coding for complement-related proteins, including inhibitors of the system, must be evaluated, as they may provide relevant information for long-term management of patients. The workup should include FH, FI, MCP, C3, FB and THBD genes. Table 6 describes protein function for these genes along with the frequency of genetic defects as determined in two large cohort studies. Complement Factor H-related genes and other rare forms of aHUS—interesting additional genes such as diacylglycerol kinase-epsilon (DGKE) or cobalamin C metabolism dysfunction (MMACHC gene)—should also be tested for.

Treatment in aHUS has been revolutionized by using complement inhibitory therapy such as Eculizumab, an anti-C5 medication [28]. Eculizumab functions by blocking the generation of proinflammatory C5a and C5b9, through binding with high affinity to C5 complement protein [29]. Although plasma exchange and i.v. immunoglobulin are often used as first measures when suspecting a MAHA, considering the underlying pathogenic mechanism in aHUS, it is this complement inhibitory medication that usually improves patients’ clinical and biological evolution.

### 4.3. TTP

We presented a case of TTP in an adolescent of 16 years. The patient also presented with severe thrombocytopenia, anemia, with normal MCV, increased reticulocyte level, decreased haptoglobin, and increased lactate dehydrogenase (LDH) level, indicating hemolysis. Positive direct Coombs’ test indicated the presence of autoimmune hemolysis. The presence of schistocytes on the peripheral blood smear, along with an ADAMTS13 activity deficiency and positive ADAMTS13 antibody, confirmed the diagnosis of TTP.

TTP is a rare type of thrombotic microangiopathy (TMA), distinguished by the occurrence of microangiopathic hemolytic anemia (MAHA), severe thrombocytopenia, and ischemic end-organ damage [30]. Plasma exchange therapy (PEX) is considered the primary treatment for TTP, while glucocorticoids are frequently used as an adjunct therapy alongside PEX [31]. Corticoid therapy is believed to accelerate the recovery of patients with TTP by inhibiting the production of ADAMTS13 inhibitors, and decreasing cytokine production and autoantibody-mediated clearance of ADAMTS13 [32]. Observational studies have demonstrated the effectiveness of corticoid therapy alone in treating TTP. However, a study of 54 patients with TTP who were treated with steroids in monotherapy reported that almost 50% of them did not respond to the treatment [33]. In comparison, a separate study that compared prednisone with cyclosporine revealed that prednisone was more effective in boosting ADAMTS13 activity and inhibiting the production of anti-ADAMTS13 antibodies [34]. In cases of refractory TTP, the addition of Rituximab to steroids and PEX can enhance the response rate. Rituximab is a monoclonal antibody that targets the CD20 antigen found specifically in the B-cell lineage [35]. Caplacizumab is a novel therapy for thrombotic thrombocytopenic purpura. It is a humanized, anti-von Willebrand factor immunoglobulin that targets the A1 domain of vWF, thus blocking its interaction with the platelet glycoprotein Ib-IX-V receptor. When administered in conjunction with standard treatment for acquired TTP, caplacizumab demonstrates efficacy in shortening the time required to normalize platelet counts and reducing the risk of recurrence [36,37]. However, caplacizumab is not approved yet for the treatment of pediatric TTP in Romania.

Main clinical particularities of TTP and aHUS are compared in Table 7.

### 4.4. COVID-19 and Thrombotic Microangiopathies

COVID-19 represents an infectious disease caused by the coronavirus SARS-CoV-2. The novel SARS-CoV-2 virus infection has had an impact on our understanding of the previously unknown interactions between the immunological mechanism and the coagulation cascade. In a pediatric patient, the risk of developing venous thromboembolism is strongly associated with the existence and advancement of underlying pathological conditions [38]. Over the course of the pandemic, novel conditions and complications have surfaced, such as multisystem inflammatory syndrome in children (MIS-C), which frequently resembles other established diseases, such as thrombotic microangiopathy or Kawasaki disease [39,40]. Infection with this virus can lead to a wide range of clinical manifestations, including pediatric TMA, as shown by a growing number of clinical case reports [16,18,19,41,42]. Although still unclear, the mechanisms behind this association might be related to loss of protection of endothelial cells and thrombocytes against complement membrane attack complexes. It is widely recognized that TMA arises due to injury to endothelial cells in small blood vessels, resulting in hemolytic anemia, thrombocytopenia and, in certain instances, organ impairment [8]. It has been proposed that SARS-CoV-2 may cause TMA by complement activation [43,44]. This can result in uncontrolled formation of the C5b9 membrane attack complex, leading to the clinical features of TMA. Soluble C5b9 (sC5b9) is a biomarker that is clinically available and has been suggested as an indicator of TMA severity [45,46]. All thrombotic microangiopathies are initiated by endothelial damage caused by the virus. Binding of SARS-CoV-2 to the ACE-2 receptors which are expressed in airway epithelial cells damages vascular endothelial cells. This leads to the release of pro-inflammatory chemo-attractants such as C3a and C5a, which recruit more leukocytes [47]. Activated leukocytes release cytokines, including IL-6, TNF, and IL-1, causing more endothelial damage and platelet aggregation. In their study, Gralinski et al. showed that SARS-CoV-2 introduced to mice caused lung damage and complement protein deposition, which was reduced when the virus was introduced to C3-deficient mice [48]. Reports have emerged that some of the vaccines against COVID-19 may be associated with vaccine-induced immune thrombotic thrombocytopenia (VITT) which is triggered by the presence of antibodies that recognize platelet factor (PF4) bound to platelets [49,50]. According to current research, it is believed that the vaccine triggers formation of neoantigens (first hit) and a subsequent systemic inflammatory response (second hit). This results in the production of anti-PF4 antibodies (platelet factor 4 antibodies) [51], which inhibit the activity of ADAMTS13, therefore unable to regulate the multimeric size of vWF., Consequently, the accumulation of ultra-sized vWF multimers can result in the formation of platelet-rich microthrombi [52]. Recent literature data suggests that there is a strong possibility that COVID-19 acts as a trigger for HUS or TTP, both of which express TMA [53]. However, it remains to be determined whether complement activation and severe endothelial injury, leading to the development of TMA, is a consequence of the typical MIS-C cytokine storm associated with COVID-19 or whether SARS-CoV-2 unmasks a primary atypical HUS or an ADAMTS 13 deficiency that have not yet been confirmed by genetic testing [54]. Phospholipase A2 (PLA2G2A) has been suggested as a proteomic biomarker for the occurrence of TMA, and MIS-C COVID-19 patients [55]. However, two extensive epidemiological studies indicate that the first occurrences of immune TTP are not heightened by SARS-CoV-2 vaccination, but may trigger acute bouts of relapses of immune TTP in individuals with a severely deficient ADAMTS13 activity who are asymptomatic [56,57]. The clinical manifestations of acquired TTP have been linked to COVID-19 in adults, but as TTP is infrequent in children, only a limited number of cases affecting children have been documented [58,59]. The cases described in our report exhibit characteristic signs indicative of a systemic thrombotic microangiopathy, including microangiopathic hemolytic anemia with consumption of platelets and dysfunction of organs. These symptoms manifested at the time or soon after COVID-19 infection that interestingly did not manifest through respiratory syndrome, but rather through digestive and neurologic involvement. This finding is in agreement with the results of the review of Zhang et al. performed on 46 cases of both children and adults who presented COVID-19-associated TMA since the beginning of the pandemic [60].

In general, the management of thrombotic microangiopathy (TMA) associated with COVID-19 is guided by the underlying pathophysiology of the disease. The primary mechanisms of TMA in COVID-19 patients are complement-mediated disease and acquired deficiency of ADAMTS13. However, COVID-19 patients may also develop TMA in association with the presence of other bacterial toxins, malignancies, or medications [61]. Nevertheless, in a vast majority of these cases, abnormalities in the complement system that serve as the initial trigger for TMA may not be identified.

## 5. Conclusions

COVID-19 caused by SARS-CoV-2 virus may act as an immunologic stimulus that triggers the onset of MAHA. At present, there are few data in the literature that discuss TTP or aHUS caused by COVID-19 infection in pediatric patients. We report on two pediatric cases presenting with aHUS and TTP following SARS-CoV-2 infection. Our observations indicate that several manifestations of MAHA can be triggered by COVID-19 infection.

## Figures and Tables

**Table 1:** Biological findings in a two-year-old male with aHUS.

Liver function	AST—143 U/L ALT—149 U/L
Inflammatory syndrome	CRP—6.45 mg/L Ferritin 737 microg/dL
Blood culture	Negative
Ionogram	Na 129 mmol/L, K 5.5 mmol/L, Alkaline reserve 14.8 mmol/L
Complement	Low C3, low C4

**Table 2:** Total complement activity analysis before treatment initiation for TTP.

Total Complement Activity
Classical pathway (hemolytic test): 45 CH50/mL (reference interval 48–103 CH50/mL)
Alternative pathway (WIELISA-Alt): 104% (reference interval 70–125%)
Complement C3: 0.71 g/L (reference interval 0.9–1.8 g/L)
Complement C4: 0.08 g/L (reference interval: 0.15–0.55 g/L)
Factor H antigen: 375 mg/L (reference interval 250–880 mg/L)
Complement factor I antigen: 89% (reference interval 70–130%)
Complement factor B antigen: 92% (reference interval 70–130%)
Anti-factor H IgG autoantibody: 16 AU/mL (reference interval < 110 AU/mL)
C1q antigen = 58 mg/L (reference interval 60–180 mg/L)
Anti-C1q IgG autoantibody = 0 U/mL (reference interval < 52 U/mL)
Haptoglobin < 0.07 g/L (reference interval 0.3–2.0 g/L)

**Table 3:** PLASMIC score calculated 6 h after admission.

Criteria and Points	
Platelet count < 30 × 10^9^ per L	1
Hemolysis variable	1
No active evidence of cancer	1
No history of stem-cell or solid-organ transplant	1
Mean Corpuscular Volume < 90 fL	0
International Normalized Ratio < 1.5	1
Creatinine < 2.0 mg/dL	1

**Table 4:** French TMA score calculated 6 h after admission.

Parameter	Points
Creatinine < 2.26 mg/dL	1
Platelet count < 30 × 10^9^/L	1
Positive anti-nuclear antibodies (ANA)	0

**Table 5:** Total complement activity analysis before treatment initiation for TTP.

Total Complement Activity
Classical pathway (hemolytic test): 64 CH50/mL (reference range 48–103 CH50/mL)
Alternative pathway (WIELISA-Alt): 105% (reference range 70–125%)
Complement C3: 1.41 g/L (reference range 0.9–1.8 g/L)
Complement C4: 0.33 g/L (reference range: 0.15–0.55 g/L)
Factor H antigen: 802 mg/L (reference range 250–880 mg/L)
Complement factor I antigen: 144% (reference range 70–130%)
Complement factor B antigen: 92% (reference range 70–130%)
Anti-factor H IgG autoantibody: 14 AU/mL (reference range < 110 AU/mL)
C1q antigen = 102 mg/L (reference range 60–180 mg/L)
Anti-C1q IgG autoantibody = 3 U/mL (reference range < 52 U/mL)
Haptoglobin < 0.07 g/L (reference range 0.3–2.0 g/L)

**Table 6:** Complement-related genes, their function and frequency of pathogenic mutations according to two large cohort studies.

Gene	Protein Function ^a^	Frequency in a Large Italian Cohort Study (*n* = 214) ^b^	Frequency in a Large French Cohort Study (*n* = 144) ^c^
**FH**	Cofactor for inactivation of C3b Inhibits the assembly of C3 and C5 convertases	24%	27%
**FI**	Cleaves and inactivates C3b	4%	8%
**MCP**	Cofactor for inactivation of C3b	7%	9%
**C3**	Initiates alternative pathway of complement	4%	8%
**FB**	Links to C3, forming C3 convertase	<1%	2
**THBD**	Complement inactivation together with FI	5%	n/a
**Mutations in more than 1 gene**		3%	4%
**No identified abnormality**		49%	34%
**Anti-FH antibodies**		3%	6%

^a^ Thurman et al., 2020 [26]; ^b^ Fremeaux et al., 2013 [23]; ^c^ Norris et al., 2010 [27].

**Table 7:** Clinical particularities of TTP and aHUS according to Scully and Goodship, 2014 [37].

**TTP**	**Characteristics**	**aHUS**
ADAMTS13 deficiency	Mechanism	Defect in complement regulation
<10%	Serum ADAMTS13 activity	>10%
Maximal incidence under 40 years	Age	Mainly childhood, also possible in adults
Moderate/severe	Thrombocytopenia	Moderate/severe
Present	MAHA	Present
Atypical	Renal failure	Typical
Typical	Neurologic symptoms	Atypical
Possible	Fever	Possible

## Data Availability

All medical records were obtained from Hospital Info Word application of our institution and can be accessed with the manager’s approval.

## Supplementary Materials

The following supporting information can be downloaded at: https://www.mdpi.com/article/10.3390/diagnostics13071228/s1, Table S1: Case reports of pediatric patients in which COVID-19 infection has been suggested as triggering factor for either TTP or aHUS; Blue shaded—patients diagnosed with TTP; Orange shaded—patients diagnosed with aHUS; IUGR—intrauterine growth restriction; ASD—atrial septal defect; VSD—ventricular septal defect; PLT—platelets; Hb—hemoglobin; CS—corticosteroids; PEX—plasma exchange; HD—hemodialysis; FFP—fresh frozen plasma; R—recovery.
