# Supplementary material for: Pitfalls of Thrombotic Microangiopathies in Children: Two Case Reports and Literature Review
Source: Diagnostics (Basel). 2023 Mar 24;13(7):1228. doi: 10.3390/diagnostics13071228 (PMC10093431; doi:10.3390/diagnostics13071228)
Supplement: Supplementary file 1 [file diagnostics-13-01228-s001.zip › diagnostics-2284212-supplementary.pdf]

Table S1. Case reports of pediatric patients in which COVID-19 infection has been suggested as triggering factor for either TTP or aHUS; Blue shaded – patients diagnosed with TTP; Orange shaded – patients diagnosed with aHUS; IUGR – intrauterine growth restriction; ASD – atrial septal defect; VSD – ventricular septal defect; PLT – platelets; Hb – hemoglobin; CS – corticosteroids; PEX – plasma exchange; HD – hemodialysis; FFP – fresh frozen plasma; R – recovery.

Reference	Underlying condition	Age and sex	PLT (/mmc)	Hb (g/dl)	Creatinine (mg/dl)	ADAMTS13 activity	ADAMTS13 inhibitor	aHUS underlying mechanism	RT-PCR-SARS-CoV-2	IgG SARS-CoV-2 antibody titer	Anti COVID-19 vaccine	Clinical features	Treatment	Evolution
**Kasturiarachi *et al.*, *2022***	Sjogren's syndrome	19 yo/ F	6000	8.5	1.17	<3%	12	N/A	Negative	elevated	N/A	Headaches, vomiting, psychosis, seizures, fever, encephalopathy, left gaze deviation, right hemiplegia, hyperreflexia upper and lower limbs, hyperkinetic movements	CS, PEX, Rituximab, antiepileptics	R
**Vorster *et al.*, *2022***	TTP (relapse)	19 yo/ F	7000	12.4	N/A	<5%	N/A	N/A	Negative	N/A	Pfizer-BioNTech mRNA vaccine dose 1	Bruising, hemoglobinuria	CS, PEX, Rituximab, Caplacizumab	R

**Vorster** ***et al., 2022***	Arrythmia of unknown etiology, previously on metoprolol	15 yo/ F	33,000	6.5	N/A	<5%	N/A	N/A	Negative	elevated	Pfizer-BioNTech mRNA vaccine dose 1	Fatigue, bruising	CS, PEX, Rituximab, FFP	R
**Vorster** ***et al., 2022***	ASD/VSD repaired; hormone suppression for precocious puberty	17 yo/ M	35,2000	11.9	N/A	<5%	N/A	N/A	Negative	elevated	N/A	Hematuria, jaundice, pallor, neurologic abnormalities	CS, PEX, Rituximab, Caplacizumab, FFP, Cyclosporine	Koate-DVI infusions twice a week
**Dominguez-Rojas** ***et al., 2022***	None	9 yo/ M	13,400	10.6	1.23	54%	N/A	N/A	Negative	elevated	N/A	Fever, abdominal pain, generalized edema, erythema	CS, PEX, Rituximab, FFP, Immunoglobulin	Died
**Kirpalani** ***et al., 2022***	Anxiety, iron deficiency	14 yo/ F	<10000	6.3	N/A	<1%	N/A	N/A	Negative	negative	Pfizer-BioNTech mRNA vaccine dose 1	Fatigue, headache, confusion, bruising	CS, PEX, Rituximab, Caplacizumab	R
**Hamza et al., 2022**	None	11 yo, M	26,7000	6.80	6.30	N/A	N/A	N/A	Positive	N/A	N/A	Anasarca, papular skin rash, hematuria,	CS, antibiotics, amlodipine, furosemide	R

											shortness of breath		
**Khandelwal *et al.*,2022**	None	4 yo, M	12,000 0	3.5 0	1.30	N/A	N/A	Anti-Factor H antibodies	Negativ e	elevate d	N/A	Anasarca, pallor, hypertension, fever 21 days prior	CS, PEX, HD, i.v. cyclophosphami de	R
**Khandelwal *et al.*,2022**	aHUS (relapse)	12 yo, M	55000	8.3 0	2.40	N/A	N/A	Anti-Factor H antibodies	Positive	elevate d	N/A	Abdominal pain, vomiting, fever 10 days prior	CS, PEX, HD mycophenolate mofetil	R
**Khandelwal *et al.*,2022**	aHUS (relapse)	13 yo, M	60000	6.9 0	3.10	N/A	N/A	Anti-Factor H antibodies	Negativ e	elevate d	N/A	Abdominal pain, vomiting, hypertension, fever 8 days prior	CS, PEX, HD mycophenolate mofetil	R
**Khandelwal *et al.*,2022**	aHUS (relapse)	7 yo, M	35000	4.8 0	3.60	N/A	N/A	Anti-Factor H antibodies	Negativ e	elevate d	N/A	Abdominal pain, oliguria, hypertension, fever 10 days prior	CS, PEX, HD mycophenolate mofetil	R
**Khandelwal *et al.*,2022**	None	10 yo, F	32000	5.8 0	7.60	N/A	N/A	Anti-Factor H antibodies	Negativ e	elevate d	N/A	Oliguria, pallor, hypertension, fever 10 days prior	CS, PEX, HD i.v., cyclophosphami de	Dialysis

**Dalkıran *et al.*,2021**	None	3 yo, F	62000	5.40	1.90	> 5%	N/A	Negative for CFH, CFI, other genetical alternative complement pathway components	Positive	N/A	N/A	Fever, respiratory distress, hypertension	CS, PEX, HD, furosemide, albumin, antibiotics, i.v. immunoglobulin	R
**Searcy et al., 2022**	None	2 yo, F	51000	5.1	0.71	>100%	N/A	Unremarkable	Positive	N/A/	N/A	Bloody diarrhea, anasarca, jaundice	Supportive care	R
**Alizadeh *et al.*,2021**	IUGR, microcephaly, postnatal embolic, diabetic ketoacidosis	16 months-old, M	< 10.000	< 7	0.39	Normal	N/A	Negative for anti-Factor H antibodies	Positive	N/A	N/A	Tachypnea, hypertension, edema, nephrotic-range proteinuria	Eculizumab, calcium-blockers, insulin	R
